# Addressing the Neuroprotective Actions of Coffee in Parkinson’s Disease: An Emerging Nutrigenomic Analysis

**DOI:** 10.3390/antiox11081587

**Published:** 2022-08-16

**Authors:** Lai Kuan Lee, Nur Anis Raihana Mhd Rodzi

**Affiliations:** Food Technology Program, School of Industrial Technology, Universiti Sains Malaysia, Gelugor 11800, Pulau Pinang, Malaysia

**Keywords:** caffeine, coffee, complementary medicine, gene expressions, nutrigenomic, Parkinson’s disease

## Abstract

Caffeine is one of the predominant dietary components and psychostimulants present in coffee, a widely appreciated beverage. Corroborating epidemiological and laboratory evidence have suggested an inverse association between the dietary intakes of coffee and the risk of Parkinson’s Disease (PD). Growing attention has been paid to the impact of coffee consumption and genetic susceptibility to PD pathogenesis. Coffee is believed to play prominent roles in mediating the gene makeup and influencing the onset and progression of PD. The current review documents a current discovery of the coffee × gene interaction for the protective management of PD. The evidence underlying its potent impacts on the adenosine receptors (A_2A_R), estrogen receptors (ESR), heme oxygenase (HO), toxicant responsive genes, nitric oxide synthase (NOS), cytochrome oxidase (Cox), familial parkinsonism genetic susceptibility loci, bone marrow stromal cell antigen 1 (BST1), glutamate receptor gene and apolipoprotein E (APOE) genotype expressions is outlined. Furthermore, the neuroprotective mechanisms of coffee for the amelioration of PD are elucidated.

## 1. Introduction

Coffee, an infusion of ground, roasted coffee beans, is the most frequently consumed beverage worldwide [1]. It is an important economic crop and the second-largest traded commodity worldwide after petroleum [2]. Coffee is known for its stimulatory effects and aroma, while the caffeine, the central nervous system stimulant, probably has a multitude of potential health effects [3]. Caffeine (1,3,7-trimethylxanthine) is the most intriguing and investigated purine alkaloid that occurs naturally in coffee beans [4]. This biologically active compound is appreciated as a neuromodulator, with documented impacts on information processing, motor behavior and cognitive performance [5]. Recently, epidemiological studies have suggested the health beneficial implications of antioxidants in coffee for protection against Parkinson’s Disease (PD) [6,7,8]. Coffee contributes to a number of bioactive compounds in daily consumption. The antioxidant property is mostly attributed to its phenolic compounds (chlorogenic acids, cafestol and kahweol), diterpenes (cafestol and kahweol), other secondary metabolites and alkaloids (caffeine and trigonellin) [9]. These compounds play pivotal roles in various ways, as they exhibit antioxidant, chemoprotective effects, anti-inflammatory and anticancer activities [10].

PD is a neurodegenerative disorder, affecting approximately 1–2% of individuals older than 60 years [11]. It is physically marked by tremor, bradykinesia, muscular rigidity and postural instability [12]. The neuropathologic changes involve the loss of pigmented dopamine-containing neurons of the substantia nigra pars compacta and the presence of Lewy bodies, the intracytoplasmic eosinophilic inclusions [13]. The underlying PD etiology is still relatively unknown. Nevertheless, a combination of genetic predisposition, advancing age and environmental exposures have emerged as the independent risk factors [12]. At present, providing symptomatic relief is the recognized therapeutic method to treat PD. Advanced pharmacological strategies have been evident to provide only a transient benefit. Thus, putative neuroprotective agents, preferably with a natural origin, have been developed as better complementary therapies with minimal side effects.

In the past few decades, coffee consumption has been proposed to demonstrate a protective effect on PD risk [14,15,16]. Although the beneficial effects of coffee have not been thoroughly discovered, its protecting influence in mediating the gene expressions of PD has received certain concern. Hence, this narrative review attempts to outline a fundamental platform to alert the specific implications of coffee for the complementary treatment of PD. The current work is aimed at suggesting a current scenario for the promising roles of coffee and caffeine for the modulation of adenosine receptors, estrogen receptors (ESR), heme oxygenase (HO), toxicant responsive genes, nitric oxide synthase (NOS), cytochrome oxidase (Cox), familial parkinsonism genetic susceptibility loci, bone marrow stromal cell antigen 1 (BST1) polymorphisms, glutamate receptor gene and apolipoprotein E (APOE) genotype expressions. The literature has been demonstrated to inform readers with key points regarding the use of caffeine and other phenolic compounds from coffee as alternative neuroprotective agents for PD.

## 2. Coffee as Potent Antioxidant

**Figure 1:**
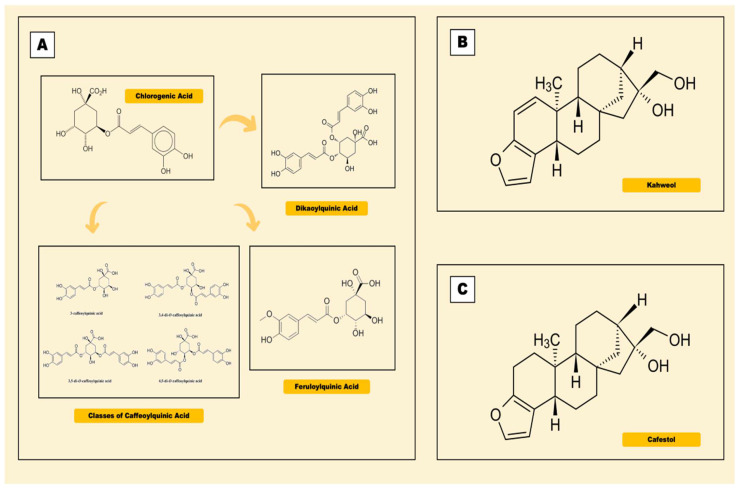
Chemical structures of phenolic compounds in coffee. (**A**) Chlorogenic acid. (**B**) Kahweol. (**C**) Cafestol.

Coffee is rich in bioactive compounds, namely caffeine, phenolic compounds, diterpenes, trigonelline and soluble fiber [9]. The most studied antioxidants present in the coffee are phenolic compounds (Figure 1), the chlorogenic acids, while cafestol and kahweol are often found in diterpenes, of which the levels are highly dependent to the degree of roasting. Table 1 shows a variety of phenolic compounds present in different types of coffee [17].

### 2.1. Coffee Roasting and Phenolic Compounds

Scientific research has revealed that standard coffee-roasting procedures modify its chemical composition. Souza and colleagues [18] detailed the effects of the roasting process on dried coffee extracts. The study reported minimal variation in total phenolic content comparing both the green and light-roast coffees. Surprisingly, an over double reduction in the amount of these compounds was reported for dark-roasted coffee, indirectly demonstrating an inverse relationship between the content of phenolic compounds with roasting degree.

In vitro studies have revealed promising results to support the role of coffee polyphenols as potential anticancer agents [18,19]. The same team assessed the antiproliferative activity of coffee extracts, together with the effects on cell cycle and apoptosis using metastatic cancer cell lines. Dark-roasted coffee depicted the lowest levels of caffeine, chlorogenic acids and caffeic acids, while both the green and light-coffee extracts showed elevated antioxidant activity and induced cytotoxicity, S-phase cell cycle arrest and apoptosis in the bone (PC-3) cell line.

### 2.2. Chlorogenic Acid

Chlorogenic acid (CGA), a family of esters formed of quinic and coffee acids, is classified into caffeoylquinic (CQA), feruloylquinic (FQA) and dikaoylquinic (diCQA) acids [20]. In humans, CGAs are hydrolyzed in the intestine with the presence of hepatic enzymes to form phenolic metabolites, primarily the derivatives of caffeic and ferulic acids [9]. High CGA content functions as a potent free radical scavenger and an inducer of the nuclear factor erythroid 2-related factor 2 (Nrf2) signaling pathway [21].

CGA has the potential to capture superoxide anions or hydroxyl radicals. An in vitro study revealed that CGA was capable of inhibiting the formation of free radicals and preventing oxidative process expansion [22]. The evidence was further confirmed by Lemos and team [23], where CGA was found superior to perform antioxidant profiles and recorded an IR50 below 7.0 μg/mL. Recently, in an effort to reveal the antioxidant effects of CGA-derived circulating metabolites, an activation of the Nrf2/ARE pathway was demonstrated among coffee drinkers [24]. Consumption of coffee phytoextracts incremented nuclear localization and HO-1 expression. The presence of quinic acid derivatives works as a powerful reactive oxygen species (ROS) scavenger against TBHP-mediated oxidative stress, with procyanidins demonstrating potent functions to suppress the intracellular inflammatory mechanisms [21].

### 2.3. Kahweol

Kahweol, a coffee diterpene molecule, is existed between 661 and 923 mg/100 g Arabica coffee bean [25]. Research suggested that kahweol reduced liver inflammation by hampering the activation of nuclear factor kappa B (NF-kβ), signal transducers and transcription factors (STAT3) [26].

Kahweol consumption is associated with HMOX1 expression, which regulates the intracellular ROS levels in human neuroblastoma cells. On the other hand, kahweol exerts anti-inflammatory effects by inhibiting macrophage cyclooxygenase-2 and nitric oxide expressions [27]. Lately, new findings suggested that pre-treatment of insulinoma (INS-1) cells with kahweol suppressed streptozotocin (STZ)-induced damage in PD. However, more studies are warranted to reveal the biological roles of kahweol against PD [28].

### 2.4. Cafestol

Cafestol is a natural diterpene that presents as fatty esters in unfiltered coffee. According to Hao and colleagues [29], cafestol exerts protecting effects by upregulating sirt1 expression in human umbilical vein endothelial cells (HUVECs) after treatment with cyclic trains. As such, cafestol may inhibit the expressions of inflammatory molecules in endothelial cells. These findings provide new insights on the molecular mechanisms underlying the effects of cafestol against inflammation-dependent disorders, such as PD.

## 3. Coffee Consumption, Gene Expressions and PD

Many epidemiological studies have linked coffee consumption with a reduced PD risk. Caffeine in coffee may exert neuroprotective effects by ameliorating the incidence, morphology and pathophysiology perspectives in PD. Recently, interactions between coffee consumption and genetic drivers of PD have become a hot topic among the scientific communities .

### 3.1. Coffee, Adenosine A_2A_ Receptor (A_2A_R) Gene Polymorphisms and PD Risk

Adenosine receptors (ARs), members of the G protein-coupled receptor superfamily [30], are highly expressed in the basal ganglia [31]. The striatum strongly expresses the adenosine A2a receptor (A_2A_Rs), which plays important roles in the regulation of dopaminergic transmission [32]. Four single-nucleotide polymorphisms (SNPs) have been identified for the A_2A_R gene: the rs5751876, rs71651683, rs3032740 and rs5996696 [33]. Epidemiological studies have revealed that caffeine is an A_2A_R antagonist and a putative functional genetic variant of the A_2A_R might mediates caffeine–PD association [34].

#### 3.1.1. Human Study

A preliminary human study examining the interaction between coffee consumption, A_2A_R variability and the risk of PD was investigated by Tan and co-workers [35] (Table 2). The multivariate regression model confirmed the protective effect of one daily cup of coffee consumption alone (up to 10 years), as this reduced PD risk by 8%. The result implied that the protective effect of coffee intake in PD was independent of the A_2A_ 2592C > Tins (rs3032740) polymorphism. The lack of evidence for the A_2A_R gene suggested that PD may be regulated differently from other caffeine-induced neurodegenerative disorders.

Two years later, a nested case-control study was undertaken in Rochester, United States, to investigate the role of A_2A_R and cytochrome P450 1A2 (CYP1A2) gene polymorphisms and its joint interaction with lifetime coffee drinking pattern in relation to PD susceptibility [36]. Two SNPs within the A_2A_R genes (rs5751876 and rs3032740) and two CYP1A2 genes (rs35694136 and rs762551) were genotyped using a chip-based platform. The study did not support the notion that coffee drinking alone, or of the genetic variants, had an effect on PD susceptibility.

The results from the Parkinson’s Epidemiology and Genetic Associations Studies in the United States (PEGASUS) were compiled using both the DNA and risk-factor data from five population-based case-control studies [37]. Four A_2A_R (rs5751876, rs71651683, rs3032740 and rs5996696) and three CYP1A2 (rs762551, rs2472304 and rs2470890) polymorphisms were genotyped. This finding indicated that only CYP1A2 variants appeared to show neuroprotective effects of coffee on PD risk. Discovery of the molecular mechanisms on how coffee is able to affect the gene expressions would contribute to the preventive treatment of PD.

#### 3.1.2. In Vivo Study

The first in vivo study investigating the impact of A_2A_R, CYP1A2 and dopamine transporter (DAT) gene expressions by measuring their levels in 1-methyl 4-phenyl 1,2,3,6-tetrahydropryridine (MPTP)-treated mouse striatum was conducted by Singh et al. [46]. Results revealed that administration of caffeine partly protected against MPTP-induced neurologic changes, modulated MPTP-mediated expression and catalytic activity of CYP1A2, A_2A_R and DAT. These findings emphasized that CYP1A2, A_2A_R and DAT partially contributed to the caffeine-mediated neuroprotective effect [47].

### 3.2. Caffeine, Estrogen Receptor (ESR) Genes and PD Risk

Estrogen receptor alpha (ESR1) and estrogen receptor beta (ESR2) are the nuclear transcription factors (NTFs) involved in the regulation of many complex physiological functions. The ESRs coupled with the administration of low and moderate doses of caffeine showed neuroprotective effects against PD, particularly in women [54,55].

#### Human Study

Palacios and colleagues [38] investigated the associations between caffeine-metabolizing genes ((CYP1A2 and N-acetyltransferase 2 (NAT2)), estrogen receptors (ESR1 and ESR2), caffeine intake and hormone replacement therapy (PMH) use and the risk of PD. The study highlighted that female carriers of the rs762551 polymorphism of CYP1A2 were at an increased risk for PD. However, the roles of caffeine intake, estrogen status and risk of PD deserve further justification.

### 3.3. Kahweol, Heme-Oxygenase-1 (HO-1) and Oxidative Stress in PD

Heme oxygenase (HO) is a family of cytoprotective and rate-limiting enzymes, which catalyzes the degradation of heme [56]. HO-1 is induced by nitrosative and oxidative stress against cellular injury and disease [57]. An examination of the protective mechanisms in PD illustrated that HO-1 expression was capable of protecting the cortical and dopaminergic neurons from MPP^+^-induced oxidative insult [58]. Increasing interest has been devoted to discovering the interconnection between HO-I expressions, together with environmental exposure in affecting PD risk.

#### In Vitro Study

The protective effect of coffee diterpene kahweol in vitro was evaluated by determining its influence on HO-1 expressions in PD etiology. Human neuroblastoma SH-SY5Y cells were treated with kahweol before being incubated with neurotoxin 6-hydroxydopamine (6-OHDA) for a subsequent 24 h [51]. Cell viability was measured using a cell proliferation assay and the TUNEL assays were applied to assess the protective effects of kahweol against 6-OHDA-induced apoptosis. ROS generation, HO-1 expressions, Nrf2 nuclear translocation and the PI3K pathway induction were assessed. Results showed that kahweol-pretreated SH-SY5Y cells significantly reduced 6-OHDA-induced ROS and caspase-3 activation. In addition, kahweol activated the induction of Nrf2 and HO-1 expressions via the phosphatidylinositol 3-kinase (PI3K) and p38 pathway.

Similarly, kahweol also promoted mitochondrial protection and decreased the level of oxidative stress markers [52]. This useful phenolic compound decreased the methylglyoxal-induced prevented mitochondria-related bioenergetics decline, loss of mitochondrial membrane potential and suppressed production of ROS and reactive nitrogen species (RNS) [53].

### 3.4. Caffeine, Toxicant Responsive Genes and PD

#### In Vivo Study

In 2008, Singh and team investigated the roles of nicotine and caffeine on toxicant responsive gene expression in MPTP-induced PD [48]. Caffeine (20 mg/kg) or nicotine (1 mg/kg) were administered to mice for eight weeks, followed by MPTP (20 mg/kg) + nicotine or caffeine in the following four weeks. Ribonucleic acid (RNA) was extracted from the striatum and polymerase chain reaction (PCR) amplification was conducted for 7-ethoxyresorufin O-deethylase (CYP1A1), *p*-Nitrophenol O-hydroxylase (CYP2E1), glutathione-S-transferase-ya (GST-ya), glutathione-S-transferase-yc (GST-yc), glutathione S-transferase 4, alpha 4 (GSTA4-4), vesicular monoamine transporter-2 (VMAT-2) and glyceraldehyde 3-phosphate dehydrogenase (GAPDH). The activities of CYP1A1, CYP2E1 and GST were measured, while the expression of VMAT-2 was confirmed by Western blot analysis. High-performance liquid chromatography (HPLC) was used to measure the striatal dopamine and MPP^+^ levels. The result implied that MPTP significantly attenuated CYP1A1 and VMAT-2, and augmented CYP2E1, GST-ya, GST-yc and GSTA4-4 expressions and activities. Hence, the reduced neurotoxicity is probably attributed to the capabilities of caffeine or nicotine to restore most of the MPTP-induced alterations.

Administration of caffeine or nicotine was associated with a higher degree of tyrosine hydroxylase immunoreactivity in the substantia nigra as compared with the MPTP-treated mice [46]. Caffeine- and nicotine-treated mice restored the gene expressions involved in oxidative stress, apoptotic cell death, protein modification, cell cycle regulation and mitochondrial dysfunction in MPTP-exposed mice. The findings provided a platform to reveal that despite many differences, both caffeine and nicotine share some common pathways of neuroprotection in PD.

### 3.5. Caffeine, Nitric Oxide Synthase (NOS) Gene Polymorphisms and PD Risk

Nitric oxide (^•^NO) is enzymatically synthetized from L-arginine (L-Arg) by three NO synthase isoforms, iNOS (NOS2), eNOS (NOS3) and nNOS (NOS1) [59]. It has been reported that the expressions of NOS1 and NOS2, but not NOS3, are involved in PD neurodegenerative pathogenesis [60]. In fact, NOS has been found to affect sleep stages [61] and modulate the L-DOPA-induced dyskinesias in PD [62].

#### Human Study

In the United States, the relationship between NOS gene polymorphisms, caffeine intake and PD risk was first investigated in 2008 [39]. As such, 163 cases (both sporadic and familial PD) and 178 relatives and other controls were recruited, and information about caffeine consumption, cigarette smoking and pesticide exposures was obtained. The NOS1, NOS2A and NOS3 genotyping were performed using the TaqMan allelic discrimination assay. Generalized estimating equations were used to detect the gene–environment interactions. NOS1 SNPs and the NOS2A SNPs were associated with earlier-onset families with sporadic PD. Pairwise analyses revealed a significant inverse interaction between caffeine consumption and the NOS2A rs944725. The study suggested that NOS2A rs944725 is a genetic susceptibility factor for PD, particularly among the low-caffeine consumers.

Extracellular adenosine is vital for the stimulation of A_2_A receptors, upregulating microglial activation and iNOS expression through p38 and ERK1/2 MAP kinase activities [49]. As such, caffeine functions as an antagonist of A1 and A2A adenosine receptors and it is related to a decreased risk for PD and other cases of neurodegenerative-disorder-derived neuroinflammation [63].

### 3.6. Coffee/Caffeine Consumption, Familial Parkinsonism Genetic Susceptibility Loci and PD Risk

Recent advances in genetics have revealed a number of common variants of susceptible familial genes in sporadic PD. Leucine-rich repeat kinase 2 (LRRK2) is located at the chromosome 12q12 and its mutations may explain the cause of autosomal dominant PD in the advanced-age group [64,65]. Meanwhile, genotyping studies have confirmed that two haplotypes of microtubule-associated protein tau (MAPT), the H1 and H2, are prevalent among in Caucasians. However, population-based studies have suggested that specific MAPT H1 subhaplotypes are preferentially associated with PD [66]. The identification of novel gene association may help to form data pooling to further unravel the mechanisms by which genes may confer PD risk.

#### Human Study

From this perspective, Gao et al. [40] explored the interactions between caffeine intake and 10 genome-wide association studies (GWAS) on SNPs at or near the alpha-synuclein (SNCA), MAPT, LRRK2 and human leukocyte antigen (HLA) loci in PD. No significant interaction of caffeine intake with the studied gene expressions was reported in this large population study. However, a combined exposure of caffeine intake and smoking exhibited significant interactions with rs2896905 at SLC2A13 locus, near the LRRK2. The study provided some preliminary evidence to confirm that among non-smokers with low-caffeine intake, a 35% higher PD risk was observed for each A allele. Nevertheless, a 32% lower risk among smokers with high-caffeine intake was reported. Additional research is required to explore the possible convincing interaction of LRRK2 with caffeine intake and smoking.

A year later, Chung and his co-workers [41] evaluated the roles of environmental exposures and 18 variants (16 SNPs and two variable-number tandem repeats) in SNCA, MAPT and LRRK2, where 1098 PD cases and 1098 matched controls were compared. A significant pairwise interaction was observed between coffee drinking and MAPT H1/H2 haplotype (rs16940806). However, the association remained not significant after Bonferroni correction, indicating that independent studies are needed to replicate the finding.

### 3.7. Caffeine, Cytochrome Oxidase (Cox) Expressions and PD

Cytochrome oxidase (Cox) is a mitochondrial membrane integral protein responsible for the catalysis process to generate adenosine triphosphate (ATP) [67]. Cox is functioning to regulate proton pumping efficiency, ATP and ROS production, which, in turn, affects cell signaling, activating programmed cell death cascade and mediating oxidative stress [68,69].

#### In Vivo Study

The mechanism of action for caffeine to stimulate the expression of Cox in a sexually dimorphic manner in PD was examined using C57B6/S129 mice, which received intraperitoneal injections, caffeine or the A_2A_ agonist CGS21680 [50]. The results suggested that upregulation of Cox activity by caffeine explained one of the several sexually dimorphic mechanisms that protect the brain from PD.

### 3.8. Caffeine Intake, Bone Marrow Stromal Cell Antigen 1 (BST1) Polymorphisms and Sporadic PD

Bone marrow stromal cell antigen 1 (BST1) is a NAD-metabolizing ectoenzyme and a glycosylphosphatidylinositol-anchored glycoprotein located at human chromosome 4 (4p15) [70]. In a GWAS study conducted among the Japanese population, BST1 was first discovered to be associated with PD and SNPs rs11931532, rs12645693, rs4698412 and rs4538475 were identified as risk factors for sporadic late-onset PD [66]. Lately, in Taiwan, BST1 rs11724635 has been indicated as a susceptible gene to interact with environmental exposures, subsequently elevating the risk of PD [71].

#### Human Study

In 2012, Miyake et al. [42], from Japan, examined the interconnection between BST1 genotype polymorphisms and the risk of sporadic PD. The BST1 polymorphisms (rs11931532, rs12645693 and rs11724635) were genotyped through a series of buccal swab DNA extractions using TaqMan SNP Genotyping Assays on the StepOnePlus machine. The study failed to verify any significant association between the BST1 SNPs and the risk of sporadic PD. However, a borderline significance was reported between the SNP rs11724635 and sporadic PD under the co-dominant and additive models. As such, larger and well-strategized investigations are needed to validate the association.

### 3.9. Coffee Consumption, Glutamate Receptor Gene Polymorphisms and PD Risk

Glutamate receptor gene (GRIN2A) is well known for the regulation of excitatory neurotransmission in the brain region, controlling movement and behavior [72]. A genome-wide gene–environment study was conducted to ascertain the role of GRIN2A as a PD modifier gene via interaction with coffee [43]. As such, 1458 PD patients and 931 controls registered under the NeuroGenetics Research Consortium (NGRC) participated in the study, where both the lifetime caffeinated-coffee consumption and genome-wide data were available. The study inferred that heavy coffee drinkers with rs4998386_CC and rs4998386_TC genotypes showed 18% and 59% lower risk for PD, respectively.

#### Human Study

In a more recent study, to examine the association between GRIN2A_rs4998386 and PD within a homogenous southeast Sweden cohort, PD protectiveness of caffeine intake was reported of near significant [44]. The team also found joint effects of GRIN2A_ rs4998386 genotypes and caffeine intake. Heavy caffeine intake with CC genotype reduced PD risk by 47%, while having GRIN2A_rs4998386_T allele with light caffeine intake showed a trend of protectiveness, and the combination of heavy caffeine intake and GRIN2A_rs4998386_TC genotype was associated with an overall 64% PD risk reduction. These studies demonstrated that GRIN2A might act as a new modifier gene and potential pharmacogenetic marker for PD.

### 3.10. Coffee Consumption, Apolipoprotein E (APOE) Genetic Polymorphisms and PD Risk

Apolipoprotein E (APOE) encodes three common alleles (ε2, ε3 and ε4) that can be differentiated by either arginine or cysteine in the receptor-binding region [73]. A clinical-imaging study revealed that APOE ε4 allele may increase the pathological burden in the temporal region, leading to cortical atrophy in the parahippo-campal gyrus in PD [74].

#### Human Study

McCulloch et al. [45] genotyped and classified the genetic polymorphisms of APOE ε2/ε3/ε4, repeat polymorphism (REP1) of the SNCA, MAPT H1/H2 and ubiquitin carboxy-terminal esterase L1 (UCHL1) S18Y. Likelihood ratio tests and Bayesian inference revealed significant associations between MAPT, SNCA REP1, smoking, coffee drinking and PD risk. In particular, an inverse trend was observed between coffee drinking and APOE genotype, with the most dramatic PD risk was reported among the APOE ε2 carriers. The interactive effects of coffee and APOE provide a new clue for PD pathogenesis, showing potential in novel discovery based on personalized nutrition.

## 4. Neuroprotective Mechanisms of Caffeine x Gene Interaction for the Amelioration of PD

**Figure 2:**
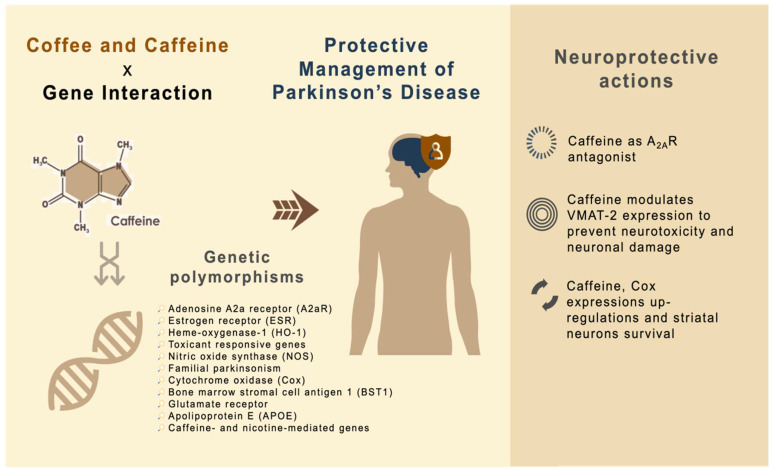
Putative mechanisms for the action of caffeine as neuroprotective agent against PD.

The promising molecular integration linking caffeine, gene expressions and PD is currently under investigation. Caffeine is the most widely investigated coffee component and benefits from regular coffee intake are typically attributed to caffeine. The proposed mechanisms reveal the neuroprotective actions of caffeine to affect gene expression, providing important insights into the development, prevention and treatment of PD (Figure 2).

### 4.1. Caffeine as A_2A_R Antagonist

A meta-analysis demonstrated that coffee intake can decrease the risk for developing PD [75]. Caffeine works as a key nonselective blocker of all four adenosine receptor subtypes: A_1_, A_2A_, A_2B_ and A_3_. Amongst these subtypes, adenosine A_2A_ receptors (A_2A_Rs) are leading to outline the beneficial actions of caffeine [76]. A_2A_R interacts antagonistically to mediate the release of gamma amino butyric acid (GABA), directly inhibiting the dopamine transmission [77,78]. The activation of the striatal adenosine A_2A_R decreases the affinity of D2 receptors for dopamine, thus, inhibiting cyclic AMP (cAMP) formation, leading to the activation of the striatopallidal/indirect pathway. In contrast, caffeine is able to antagonize adenosine A_2A_R, indirectly reduce the adenosine transmission, reverse motor deficits and prevent levodopa-induced dyskinesias [79]. Blockade of adenosine A_2A_R activity helps to protect and facilitate movement [80]. Hence, A_2A_R is the recognized therapeutic target to modulate motor symptoms in PD. Ishibashi and team [81] observed sufficient A_2A_R occupancy in PD patients through caffeine binding to striatal A_2A_R in a dose-dependent manner.

### 4.2. Caffeine Modulates VMAT-2 Expressions to Prevent Neurotoxicity and Neuronal Damage

VMAT-2 plays critical roles in chemically induced PD. The pathophysiological changes involved the alteration of VMAT-2 function or reduced VMAT-2 expression, which permits dopamine to persist in oxidative-stress-susceptible areas and induces cellular damage [82]. In this respect, two caffeine metabolites, paraxanthine and theophylline, help to increase the number of excitatory neurotransmitters of dopamine [83,84]. The expression of VMAT-2 is attenuated (following caffeine pre-treatment) at transcriptional and translational levels by MPTP [48]. Miller and colleagues [85] also proposed that VMAT-2 sequesters the dopamine and MPP^+^ inside the vesicles and protects the neuron from oxidative stress and MPP^+^-induced toxicity.

### 4.3. Caffeine, Cox Expression Upregulations and Striatal Neuron Survival

Cox is important for the regulation of oxidative metabolism. Caffeine increases the expressions of Cox1 but lowers the levels of Cox4 and Cox7c expressions in male mice in a sexually dimorphic manner. A_2A_R is taking part in mediating the stimulation process of Cox expression and activity by caffeine and the neuronal activities (such as the firing of striatal neurons within minutes) were correlated with the degree of Cox expressions [86]. Meanwhile, a study also suggested that A_2A_R-specific antagonists reduce the hyperactivity of striatopallidal neurons [87]. In a cross-sectional investigation over 5 years among PD patients, habitual coffee consumption presented changes in striatal dopamine active transporter (DAT). As A_2A_Rs are usually present in the striatum, it is believed that chronic coffee consumption observed significant changes in their striatal, suggesting potential post-synaptic colocalization in the medium spiny neurons [88]. This is because A_2A_Rs are expressed on axon collaterals of striatopallidal neurons that release GABA, as well as on axons of GABAergic interneurons of the striatum. A_2A_R signaling suppresses GABA release, resulting in an increase for the overall excitability of striatopallidal neurons. A_2A_R antagonists work oppositely by increasing GABA release but decreasing the excitability of striatopallidal neurons [89]. In addition, Mao and colleagues [90] indicated that upregulation of Cox activity by caffeine leads to increased energy metabolism in the axon terminals of dopamine neurons, hence, protecting them from oxidative damage.

## 5. The Neuroprotective Effects of Phenolic Compounds in Coffee

**Figure 3:**
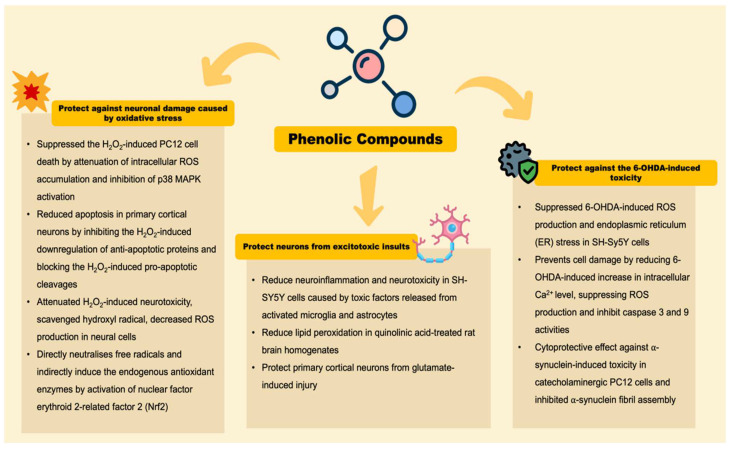
Neuroprotective effects of phenolic compounds in coffee.

The neuroprotective effects of phenolic compounds in coffee for the amelioration of PD have long been established. Coffee contains many phenolic compounds and the neuroprotective effects of these phenolic compound have been highly emphasized. The neurochemical studies conducted in recent years support the notion that phenolic compounds from coffee: (1) protect against neuronal damage caused by oxidative stress, (2) protect neurons from excitotoxic insults, and (3) protect against 6-OHDA-induced toxicity (Figure 3) [91].

## 6. Conclusions

The prescription of conventional therapeutic medications is able to offer temporarily symptomatic relief to PD patients. Advanced scientific discoveries are consistently being made to seek and propose better alternatives or complementary therapies. As a whole, coffee or caffeine-mediated neuroprotection has been demonstrated in the regulation of A_2A_R genes polymorphisms, HO expressions, toxicant responsive genes, NOS genetic variants, familial parkinsonism genetic susceptibility loci, Cox expressions, glutamate receptor and APOE gene polymorphisms. Nevertheless, the specific actions of coffee or caffeine are still not conclusive and continual investigations on the nature and biological routes of these functional compounds ought to be an immediate action. Ultimately, the discoveries and applications of these potential genomic differences would facilitate a more feasible PD study, stipulating the gene–diet interactions. In future, it is viable that trending research will be conducted to capture the discovery of nutritional intervention on gene expression into health preventive and rehabilitation strategies. Finally, the identification of genetic modifiers could assist the planning of human clinical trials for coffee or caffeine in PD.

## Figures and Tables

**Table 1:** Phenolic compound content in different types of coffee (expressed in g/100 g).

Coffee Type	Caffeoylquinic Acid (CQA)	Feruloylquinic Acid (FQA)	Dikaoylquinic Acid (diCQA)	Total Chlorogenic Acids
Green coffee	3.26–7.66	0.19–1.43	0.45–2.31	4.10–11.30
Roasted coffee	0.38–3.23	0.06–0.34	0.03–0.24	0.47–2.66
Decaffeinated coffee ^a^	5.19–6.14	0.32–0.45	0.61–0.77	6.13–7.47
Instant regular coffee	0.63–5.28	0.06–1.16	0.03–0.53	0.72–6.97
Instant decaffeinated coffee	3.33–4.73	0.60–0.84	0.17–0.28	4.10–5.85

^a^ For Arabica coffee.

**Table 2:** Neuroprotective actions of coffee in PD genetic polymorphisms.

Genetic Polymorphisms	Reference	Genes/Cells of Interest	Results
Adenosine A_2A_ Receptor (A_2A_R)	[35]	1976T/T and 2592Tins/Tins genotypes	Independent coffee-PD association of the A_2A_ 2592C > Tins (rs3032740) polymorphism
	[36]	Cytochrome P450 1A2 (CYP1A2)	≠Coffee-PD associations
	[37]	Four A_2A_R (rs5751876, rs71651683, rs3032740 and rs5996696) and three CYP1A2 (rs762551, rs2472304 and rs2470890)	Strong coffee-PD association among CYP1A2 variant allele rs762551 and rs2470890
Estrogen receptor (ESR) genes	[38]	Estrogen receptor alpha (ESR1), Estrogen receptor beta (ESR2)	↑ PD risk among female with rs762551 polymorphism of CYP1A2
Nitric Oxide Synthase (NOS)	[39]	NOS2A rs944725	Significant inverse interaction between caffeine consumption and the NOS2A rs944725
Familial Parkinsonism genetic susceptibility loci	[40]	10 genome-wide association studies (GWAS) SNPs at or near the alpha-synuclein (SNCA), MAPT, LRRK2, and human leukocyte antigen (HLA) loci	≠ significant interactions of caffeine intake with several SNPs at or near the SNCA, MAPT, and HLA loci
	[41]	SNCA, MAPT and LRRK2	Significant pairwise interaction has been observed between coffee drinking and MAPT H1/H2 haplotype (rs16940806)
Bone marrow stromal cell antigen 1 (BST1)	[42]	BST1 SNPs rs11931532, rs12645693, and rs11724635	≠ significant associations between BST1 SNPs rs11931532, rs12645693, and rs11724635 and the risk of sporadic PD
Glutamate receptor gene (GRIN2A)	[43]	rs4998386	Significant interactions from rs4998386 and the neighboring SNPs in GRIN2A
	[44]	Glutamate receptor gene (GRIN2A) rs4998386	Heavy caffeine intake & GRIN2A_rs4998386_TC genotype was associated with a ↓ 64% risk reduction Strong significant GRIN2A_rs4998386 genotype ∗ caffeine interaction
Apolipoprotein E (APOE)	[45]	Genetic polymorphisms of APOE ε2/ε3/ε4, repeat polymorphism (REP1) in the promoter region of the SNCA, MAPT H1/H2 and ubiquitin carboxy-terminal esterase L1 (UCHL1) S18Y	Inverse association between coffee drinking and APOE genotype Most dramatic PD risk in APOE ε2-carriers
Adenosine A_2A_ Receptor (A_2A_R)	[46,47]	CYP1A2 and dopamine transporter (DAT)	Coffee-PD partially associated by CYP1A2, A_2A_R and DAT
Toxicant responsive genes	[48]	7-ethoxyresorufin O-deethylase (CYP1A1), *p*-Nitrophenol O-hydroxylase (CYP2E1), glutathione-S-transferase ya (GST-ya), glutathione-S-transferase yc (GST-yc), glutathione S-transferase alpha 4 (GSTA4-4), vesicular monoamine transporter-2 (VMAT-2) and glyceraldehyde 3-phosphate dehydrogenase (GAPDH)	MPTP significantly attenuated CYP1A1 and VMAT-2, and augmented CYP2E1, GST-ya, GST-yc and GSTA4-4 expressions and activities
Nitric Oxide Synthase (NOS)	[49]	NOS2A rs944725	↑ microglial activation and iNOS expression by boosting p38 and ERK1/2 MAP kinase activities
Cytochrome oxidase (Cox) expressions	[50]	Cytochrome oxidase 1 (Cox1), cytochrome oxidase 4 (Cox4), cytochrome oxidase 7c (Cox7c)	↑ Cox1, Cox4 and Cox7c in the striatum of male mice, but not in female mice after receiving a single dose of caffeine ↑ Cox7c mRNA expression in the striatum and in PC-12 cells
Heme oxygenase-1 (HO-1)	[51]	Human neuroblastoma SH-SY5Y cells (Pretreatment of SH-SY5Y cells with kahweol)	Pretreatment of SH-SY5Y cells with kahweol significantly reduced 6-OHDA-induced generation of ROS and caspase-3 activation. Protects against 6-OHDA-induced neuronal cell death. Kahweol activated the induction of Nrf2 and HO-1 expression via the phosphatidylinositol 3-kinase (PI3K) and p38 pathway
	[52,53]	Human neuroblastoma SH-SY5Y cells	↑ mitochondrial protection in SH-SY5Y cells exposed to H_2_O_2_ ↓ oxidative stress markers ↓ production of ROS

## Data Availability

Not applicable.
